# Case Report: Ultrasound-guided fine-needle aspiration for parathyroid cyst

**DOI:** 10.3389/fradi.2025.1694006

**Published:** 2025-10-23

**Authors:** A. Serblin, R. Valcavi

**Affiliations:** ^1^ Department of Otolaryngology, Head and Neck Surgery Unit, University Hospital of Ferrara, Ferrara, Italy; ^2^ Department of Endocrinology, The Endocrine & Thyroid Clinic, Reggio Emilia, Italy

**Keywords:** parathyroid cysts (PCs), cystic thyroid nodule, parathyroid hormone (PTH), fine-needle aspiration (FNA), thyroid ultrasound (US)

## Abstract

A 55-year-old man was referred to our Department with a cystic lesion in the lower right lobe of the thyroid, incidentally discovered on ultrasound. The mass measured 52.1 × 55.3 × 66.8 mm, with a volume of 93.2 mL, and caused significant tracheal indentation with contralateral deviation. The patient was asymptomatic and did not have dysphagia, hoarseness or dyspnoea. Ultrasound-guided fine-needle aspiration of the lesion yielded a clear, “rock-water” fluid. Biochemical analysis of the aspirate revealed elevated parathyroid hormone (PTH), leading to a diagnosis of a parathyroid cyst (PCs). This case highlights the importance of considering PCs in the differential diagnosis of large cystic neck masses, particularly when they mimic thyroid nodules. We report on this case and discuss the diagnostic challenges and management strategies for this rare condition.

**Introduction:**

Parathyroid cysts (PCs) are uncommon benign neck masses, making up 1%–5% of all neck lumps and typically affecting women aged 40–60. While many cases are asymptomatic, they often present as a palpable mass in the neck, which can lead to misdiagnosis as a solitary thyroid nodule. Large cysts can cause compressive symptoms like difficulty swallowing, hoarseness, and tracheal deviation. Diagnosis involves imaging modalities like ultrasound, CT, and MRI to confirm the cystic nature of the mass. A key diagnostic step is fine-needle aspiration (FNA), where elevated parathyroid hormone (PTH) in the cyst fluid can confirm its parathyroid origin, even if blood PTH levels are normal. Treatment depends on whether the cyst is functional or causing symptoms. Options for non-functional cysts include aspiration or sclerotherapy, though recurrence is common. Surgical removal is the definitive treatment for functional cysts, symptomatic cysts, or when the diagnosis is uncertain. Minimally invasive techniques like radiofrequency ablation (RFA) and ethanol ablation (EA) are also effective, particularly for symptomatic non-functional cysts.

**Method:**

A 55-year-old male patient presented with an incidental finding of a right inferior thyroid cystic lesion measuring 52.1 mm (AP) × 55.3 mm (T) × 66.8 mm (Sag) with a volume of 93.2 mL on ultrasound examination. The patient underwent an ultrasound guided fine-needle aspiration (FNA) of the cystic formation. Approximately 90 mL of clear, “rock water”-colored fluid was extracted. To confirm the diagnosis of a parathyroid cyst, biochemical analysis of the aspirated fluid was performed. Parathyroid hormone (PTH) and thyroglobulin (Tg) levels were measured in the cyst fluid. The results showed a PTH concentration of 1,845.80 ng/L and a Tg level of 0.37 µg/L. Cytological analysis of the aspirated material revealed amorphous, acellular content. The combination of the high PTH concentration in the aspirate and the low Tg level confirmed the diagnosis of a non-functioning right inferior parathyroid cyst. A six-monthly follow-up ultrasound for 5 years was performed to assess for recurrence, which showed no evidence of fluid re-accumulation. Serum levels of calcium, PTH and vitamin D were measured, which were found to be normal both before and after the procedure.

**Results:**

In a 55-year-old male, an incidental ultrasound and fine-needle aspiration revealed a right inferior parathyroid cyst. Approximately 90 cc of clear, “rock water”-like fluid was drained, with fluid analysis confirming high parathyroid hormone (PTH) levels. Despite the cystic finding, both pre- and post-procedural plasma levels of PTH, calcium, and vitamin D remained within normal limits. This indicates a non-functioning cyst that did not disrupt systemic endocrine balance. A six-monthly follow-up ultrasound for 5 years showed no recurrence of the cystic lesion. These findings highlight that even large, PTH-rich parathyroid cysts can be non-functional and managed effectively with simple aspiration, often with no subsequent recurrence.

**Discussion:**

Based on the findings, we conclude that this case represents a non-functioning parathyroid cyst. The patient's normal plasma levels of PTH, calcium, and vitamin D both before and after the procedure confirm that the cystic lesion did not secrete hormones in a way that affected systemic metabolism. The successful management of the cyst with fine-needle aspiration and the subsequent absence of recurrence for 5 years with a six-monthly follow-up ultrasound demonstrate that this simple, minimally invasive approach can be an effective and definitive treatment for such lesions. This case report highlights that even large PCs can be non-functional and managed conservatively with excellent long-term results.

## Introduction

Parathyroid cysts (PCs) are rare clinical and histological entities, representing a small fraction of parathyroid pathology, typically 1%–3.3% of cases (1, 2). They also account for 1%–5% of all neck masses (2, 3). While they can occur at any age, they are most frequently discovered in women between 40 and 60 years old (1, 4, 5). Based on the provided sources, the embryological derivation of parathyroid cysts is primarily linked to theories concerning the formation of true cysts. Here is an illustration of the potential embryological origins of parathyroid cysts (6):

1. The Epithelial Tubule Hypothesis: One theory explaining the origin of cystic parathyroid lesions (CPLs) hypothesizes that varying numbers of epithelial tubules, arising near thymic tissue, develop into canalicular or glandular tissue layers in postnatal life (6).
2. Embryological Remnants (True Cysts): Parathyroid cysts are classified into subgroups based on their etiology, including (a) degeneration of an adenoma or hyperplastic gland, and (b) true cysts with an epithelial layer. In clinical practice, microscopic findings can support this origin: the authors reported a case (Patient 2) where the cystic wall was composed of stratified parathyroid cells uniformly lining the CPL. This finding suggested a true parathyroid cyst, potentially derived from an embryological remnant. Ippolito et al. defined “true cysts” exclusively as the non-functioning parathyroid lesions (6).
3. Other Etiological Theories (Non-Embryological): It is important to note that while embryological remnants explain the origin of true cysts, another prevalent theory for CPLs involves the degeneration of an adenoma or hyperplastic gland. For instance, in the first case reported by the authors, the pathological finding aligned with cystic degeneration due to intraglandular hemorrhage. In summary, the embryological derivation focuses on the formation of true cysts (often non-functioning) arising from epithelial tubules associated with nearby thymic tissue or other embryological remnants.

The distinction between functioning and non-functioning parathyroid cysts (CPLs) primarily rests on hormonal activity, etiology, and required treatment. Functioning Cysts (Hyperfunctioning) cause hyperparathyroidism, evidenced by elevated serum PTH and calcium levels. Diagnosis is confirmed by highly elevated PTH values in the intracystic fluid (e.g., > 5,000 pg/mL). Etiologically, they often arise from the cystic degeneration of a parathyroid adenoma. The definitive treatment for functioning CPLs is surgical excision (6).

Non-functioning Cysts are more common and present with non-specific physical features. They generally cause compressive symptoms (e.g., neck swallowing) rather than systemic hormonal issues. Histologically, non-functioning cysts are often described as pseudocysts lacking cuboidal epithelium. For non-functioning lesions, Fine Needle Aspiration (FNA) can be considered the first-line treatment (6).

Ultrasound is indicative: non-functioning cysts have no intrinsic tissue, while cystic adenomas have observable tissue. Furthermore, true cysts only contain amorphous material in cytology; cystic adenomas, if FNA is directed at observable tissue after emptying, offer a cytological examination with cuboidal cells, similar to thyroid cells, but without background colloid. It is important to empty the cyst before performing FNA for cytology: ultrasound can only detect parenchymal content after the cyst has been emptied, because the parenchyma is compressed by intracystic pressure (6, 7).

The definitive way to distinguish a parathyroid cyst (PC) from other cystic neck masses, such as a thyroid cyst, branchial cleft cyst, or thyroglossal duct cyst (TDC), is through Fine Needle Aspiration (FNA) of the cyst fluid followed by biochemical analysis for specific markers. The key distinguishing feature is the measurement of Parathyroid Hormone (PTH) levels in the aspirated cystic fluid. The measurement of i-PTH in the fluid, which will always be clear water in the case of parathyroid cysts. And with the measurement of thyroglobulin in the fluid—citrine or hemorrhagic—high in thyroid cysts and thyroglossal duct cysts and not in branchial cysts.

Clinical presentation of PCs can be highly variable. Many cases are asymptomatic and discovered incidentally during investigations for other conditions (2, 4, 8). However, PCs frequently manifest as palpable neck masses, which is the most common symptom, occurring in 41.7% of reported patients (2). Due to their location and consistency, they often mimic other cervical pathologies, particularly solitary thyroid nodules, making accurate preoperative diagnosis difficult (2, 9). When PCs reach large dimensions, they can exert a compressive effect on adjacent structures, leading to symptoms such as dysphagia (difficulty swallowing), hoarseness, dyspnoea, and tracheal deviation (2). The pathogenesis of PCs is thought to involve several mechanisms, including congenital acquisition from branchial pouch remnants, coalescence of microcysts, or cystic degeneration/hemorrhage of a parathyroid gland or adenoma (2, 3). The diagnostic workup for PCs is crucial due to their diverse presentations and the need to differentiate them from other neck masses, such as thyroglossal duct cysts, branchial cleft cysts, and thyroid adenomas or carcinomas. Ultrasound (US) is typically the first-line imaging modality, effectively confirming the cystic nature of the mass and assessing its dimensions and relationships with surrounding structures. Computed Tomography (CT) and Magnetic Resonance Imaging (MRI) provide further anatomical detail, confirm the cystic character, and evaluate the extent of compression on vital structures like the trachea and esophagus. 99mTc-sestamibi scintigraphy can be helpful for localizing hyperfunctioning parathyroid tissue, but its sensitivity is limited, and it may yield negative or equivocal results even in functional cysts, depending on the amount of secreting tissue present in the cyst wall (1, 3, 10). Fine-needle aspiration (FNA) of the cyst fluid is a key diagnostic tool. The fluid is often described as clear or “rock-water” fluid, but can also be hemorrhagic or turbid. Biochemical analysis of the aspirate for PTH levels is highly indicative of parathyroid origin; significantly elevated intracystic PTH levels confirm the diagnosis of a PCs, even if serum PTH levels are normal. The histopathological examination of the cyst wall, revealing parathyroid tissue, ultimately confirms the diagnosis. Management strategies depend on the cyst's functionality and clinical presentation (11, 12). For uncomplicated non-functional cysts, initial management may include ultrasound-guided aspiration, although recurrence is common. Sclerotherapy with agents like tetracycline or ethanol has also been used, but carries risks of fibrosis and recurrent laryngeal nerve pals. Surgical excision is a type of treatment for functional PCs, symptomatic non-functional cysts (e.g., due to compression, recurrence), or when diagnostic uncertainty persists. Minimally invasive treatments such as radiofrequency ablation (RFA) and ethanol ablation (EA) have been shown to be effective for various benign parathyroid lesions (13). In particular, EA is effective for symptomatic non-functioning parathyroid cysts (SNPC), leading to significant reductions in size and hormone levels (13). This case report highlights the critical need to include parathyroid cysts in the differential diagnosis of large cystic neck masses, especially given their ability to mimic thyroid nodules and cause compressive symptoms.

## Method

**Figure 1:**
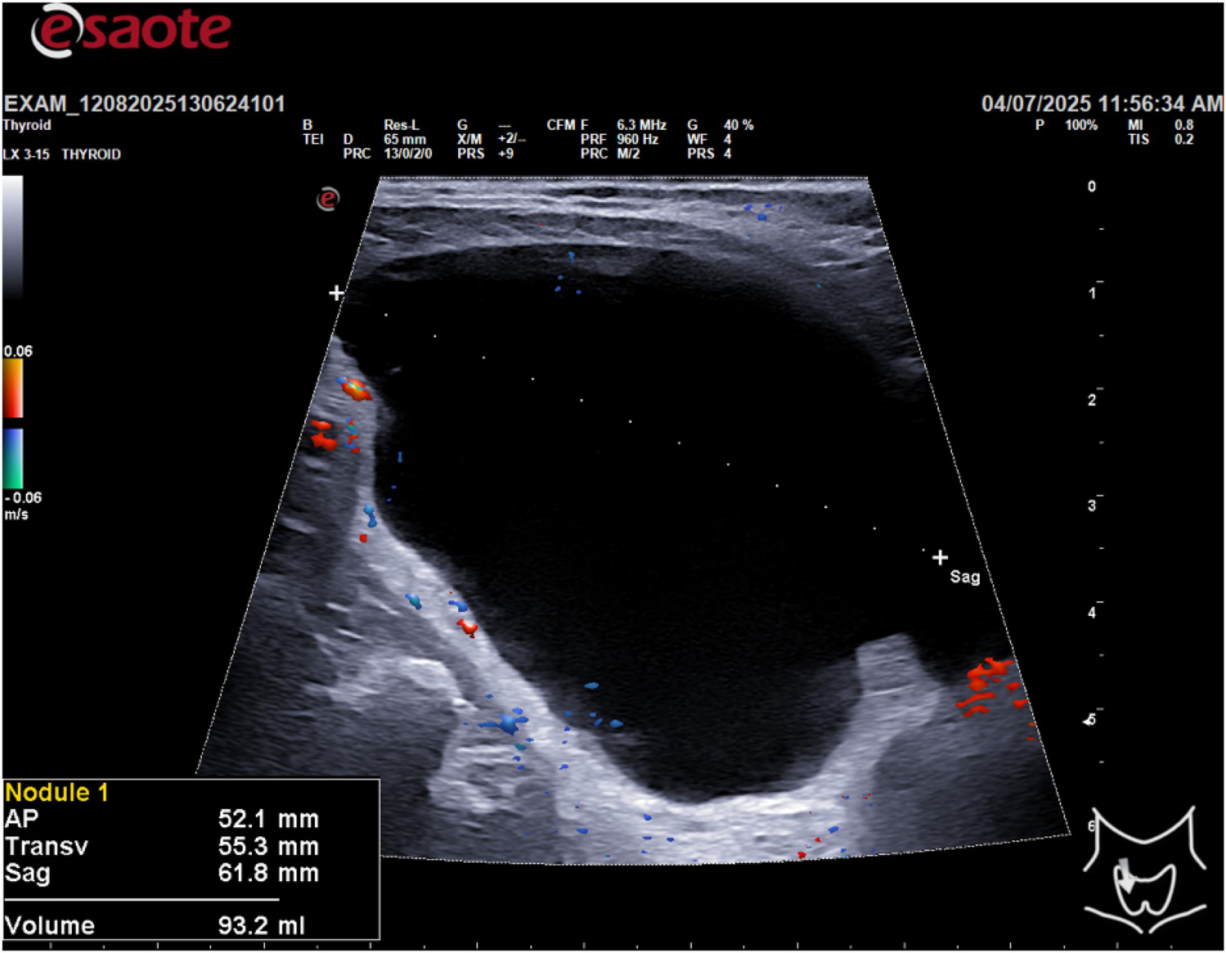
Sagittal diameter and volume of the lesion.

This study describes a single case report of a 55-year-old male with a family history of papillary thyroid carcinoma. The patient presented with an asymptomatic thyroid nodule detected on routine ultrasound. All clinical and imaging data were collected from the patient's medical records. The patient provided informed consent for the use of his data for research purposes. A thyroid ultrasound was performed using a high-frequency linear transducer (10–14 MHz). The ultrasound scan revealed a large, cystic lesion in the inferior portion of the thyroid gland with the following dimensions: AP 52.1 mm, Transverse 55.3 mm, and Sagittal 66.8 mm, for a calculated volume of 93.2 ml (Figure 1).

**Figure 2:**
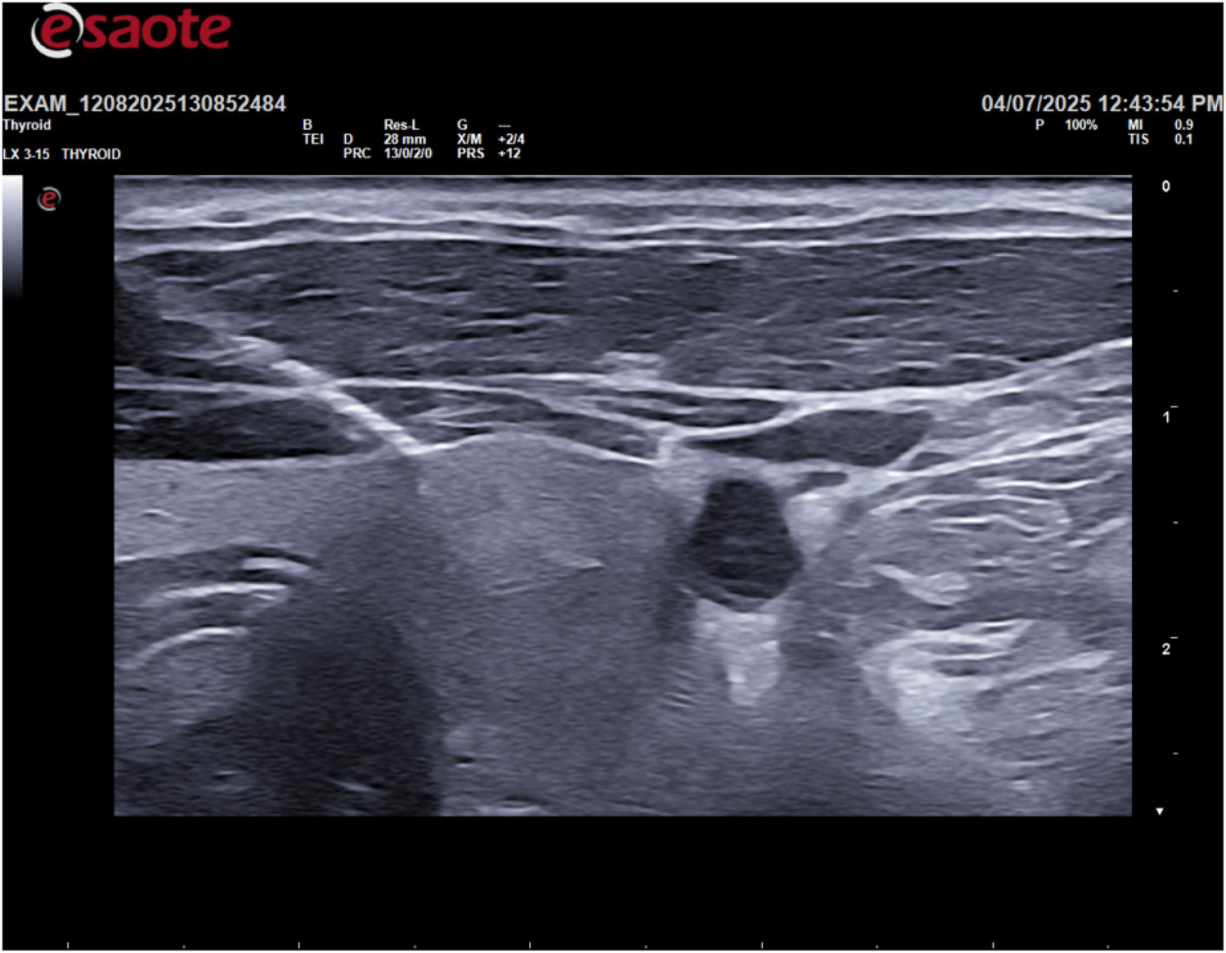
Ultrasound-guided fine-needle aspiration (FNA).

**Figure 3:**
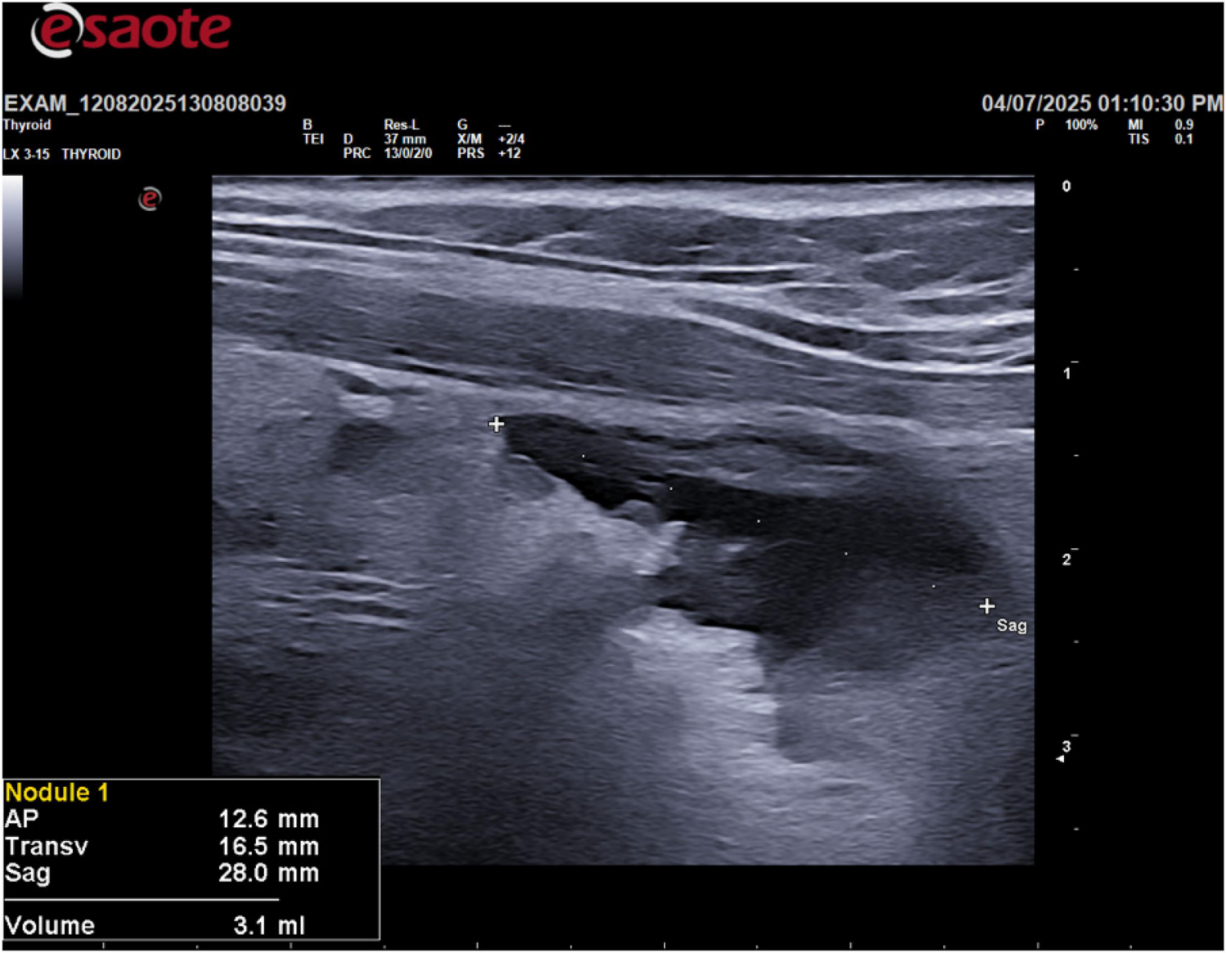
Post fine-needle aspiration (FNA) ultrasound image.

Following the ultrasound, an ultrasound-guided fine-needle aspiration (FNA) was performed on the cystic lesion (Figure 2). Approximately 90 ml of clear, “rock water"-colored fluid was aspirated (Figure 3). The cyst has been completely drained: AP 12.6 mm, Transverse 16.5 mm, and Sagittal 28 mm, for a calculated volume of 3.1 ml (Figure 3). The aspirated cystic fluid was immediately sent for biochemical analysis.

**Figure 4:**
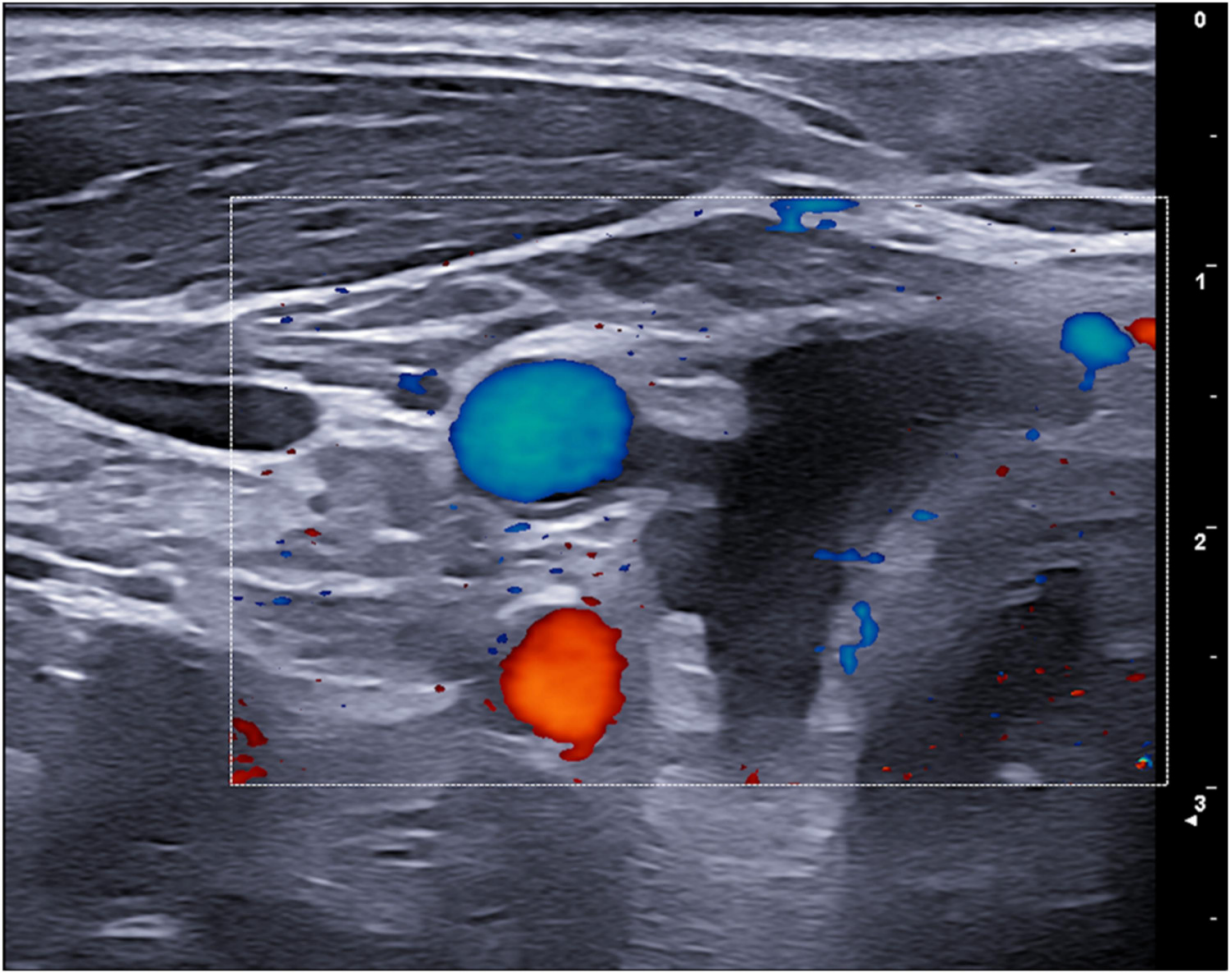
6-month ultrasound follow-up with no recurrence of cysts or cystic degeneration.

## Results

Parathyroid hormone (PTH) and thyroglobulin levels were measured in the fluid using standard laboratory assays. The results were as follows:

Aspirate Thyroglobulin: 0.37 μg/L [<55 μg/L]

Aspirate PTH: 1,845.80 ng/L [15.0 ng/L–105.0 ng/L]

Serum PTH, vitamin D, thyroglobulin, and calcium values are as follows:

PTH 49.8 ng/L (6.7–38.8)

Vit D 79.2 mmol/L (<30 deficiency)

Tg 12.2 ng/mL (1.5–25 ng/mL)

Calcium 9.5 mg/dL (8.7–10.4)

Ionized calcium 1.26 mmol/L (1.15–1.35)

Cytological examination of the aspirate showed amorphous acellular material. In addition, serum levels of PTH, calcium, and vitamin D were measured both before and after the FNA procedure to assess for any changes. The patient was monitored by ultrasound and blood tests with a 6-month and yearly follow-up extended to 5 years. No further intervention was required.

## Discussion

This case report describes an interesting and rare case of a non-functional, non-PTH-secreting parathyroid cyst, which was successfully treated with a single ultrasound-guided fine-needle aspiration (FNA). The definitive way to distinguish a parathyroid cyst (PC) from other cystic neck masses, such as a thyroid cyst, branchial cleft cyst, or thyroglossal duct cyst (TDC), is through Fine Needle Aspiration (FNA) of the cyst fluid followed by biochemical analysis for specific markers. The key distinguishing feature is the measurement of Parathyroid Hormone (PTH) levels in the aspirated cystic fluid. The measurement of i-PTH in the fluid, which will always be clear water in the case of parathyroid cysts. And with the measurement of thyroglobulin in the fluid—citrine or hemorrhagic—high in thyroid cysts and thyroglossal duct cysts and not in branchial cysts.

At our center, we only perform iPTH and thyroglobulin tests on cysts containing clear water. Thyroglobulin is used to rule out a thyroid cyst. Cytological examination is not sufficient.

In the event of cyst recurrence, radiofrequency thermal ablation is possible as a definitive solution to recurrence (14). If the parathyroid cyst is not treated, possible complications may include: compression symptoms (hoarseness, dysphagia, dyspnea) and hyperparathyroidism in the case of a cystic adenoma.

The six-month follow-up extended to five years showed no recurrence of the disease. This finding highlights the long-term effectiveness of a single FNA procedure in treating such lesions. In conclusion, this case underscores the importance of considering a parathyroid cyst in the differential diagnosis of cystic neck masses, even when serum PTH levels are normal. It also demonstrates the efficacy of ultrasound-guided FNA as a safe, effective, and minimally invasive diagnostic and therapeutic tool for these rare lesions.

## Data Availability

The raw data supporting the conclusions of this article will be made available by the authors, without undue reservation.
